# Physical activity negatively associated with symptomatic dizziness: a cross-sectional study

**DOI:** 10.1186/s12889-025-22808-y

**Published:** 2025-04-22

**Authors:** Jiqiang Zhu, Xianfeng Li, Dongxia Sun, Kuo Geng, Mengcui Wei, Jia Liu, Jing Lu

**Affiliations:** 1 Neurology, Beijing HuaSheng Rehabilitation Hospital, Beijing, 100071 China; 2 Traditional Chinese Medicine, Xianghe County Traditional Chinese Medicine Hospital, Langfang, 065400 China

**Keywords:** Physical activity, Symptomatic dizziness, NHANES

## Abstract

**Background:**

Dizziness is a prevalent complaint in clinical settings; however, its relationship with physical activity remains unclear. This cross-sectional study aimed to explore the link between physical activity levels and symptomatic dizziness in a cohort of adult participants.

**Methods:**

We used data from the 1999-2004 National Health and Nutrition Examination Survey (NHANES) in the United States. Activity and dizziness data were obtained using physical activity and balance questionnaires. The participants were divided into three subgroups, Group 1 (sedentary: almost no engagement in any form of aerobic or anaerobic exercise in the past 30 days), Group 2 (moderate: at least 10 minutes of moderate-intensity physical activity in the past 30 days, which results in light perspiration or a minor-to-moderate rise in heart and breathing rates), and Group 3 (vigorous: engaging in at least 10 minutes of vigorous-intensity exercise over the last 30 days, which leads to substantial sweating or a pronounced increase in both breathing and heart rates). Multivariable logistic regression and stratified interaction analyses were used to examine the association between physical activity and symptomatic dizziness.

**Results:**

A total of 6815 participants were enrolled, comprising 3446 males (50.6%) and 3369 females (49.4%), with a median age of 60.6±13.3 years. Our study revealed a negative association between physical activity and the prevalence of symptomatic dizziness after multivariate adjustment (Group 2, OR 0.76, 95% CI 0.66-0.87, *p*<0.001; Group 3, OR 0.76, 95% CI 0.64-0.90, *p*=0.001). Further exploratory subgroup analysis showed no statistical significance (all *P*-values for interaction were greater than 0.05).

**Conclusion:**

The study found that physical activity is negatively associated with the prevalence of symptomatic dizziness in the US adult population.

## Introduction

Dizziness is a common self-reported symptom, with a lifetime prevalence ranging from 15% to 36% [1]. Chronic dizziness and balance disorders are well known to not only severely diminish individuals’ quality of life but also increase the risk of falls and mortality. Therefore, effective management of these symptoms is particularly crucial, as it helps prevent further complications and ensures that patients can safely and comfortably engage in their daily activities. Similar to other chronic conditions such as heart disease, cancer, and diabetes, dizziness and balance disorders may require ongoing medical intervention [2]. The resulting financial burden on healthcare is substantial, with an estimated annual cost of $48.1 billion associated with dizziness [3], imposing a significant economic strain on the healthcare system and a heavy personal burden on patients. Dizziness is a common symptom with a wide range of potential causes, including benign paroxysmal positional vertigo and more serious conditions such as stroke and brain tumors [4]. Patients may experience various manifestations of symptomatic dizziness, including vertigo, lightheadedness, imbalance, and unsteadiness [5, 6]. Therefore, it is essential to identify the daily habits that may be linked to symptomatic dizziness.

Physical activity is an integral part of daily life, and research has consistently demonstrated its potential to lower blood pressure and enhance cardiovascular health [7]. Engaging in regular exercise can improve heart function, mitigate insulin resistance and inflammation within the body, and promote vasodilation, thereby reducing blood vessel resistance [8]. Additionally, weight loss, anxiety reduction, and depression alleviation often associated with physical activity contribute to the lowering of blood pressure [9], making it a holistic approach to well-being and health management.

## Methods

### Study participants

This cross-sectional study used data from the National Health and Nutrition Examination Survey (NHANES) conducted by the Centers for Disease Control and Prevention from 1999 to 2004. NHANES aims to assess the health and nutritional status of the non-institutionalized U.S. population using a stratified multistage probability sampling method. It gathers comprehensive demographic and health information through home visits, screenings, and laboratory tests at Mobile Examination Centers. The survey is authorized by the National Center for Health Statistics (NCHS) Institutional Review Board, and all participants provide written informed consent prior to their participation. Secondary analyses do not require additional approval from the Institutional Review Board. The NHANES data are accessible through the NHANES website (accessed on May 1, 2024). Our study included individuals over the age of 20 who completed the interviews. Pregnant women and individuals with missing data regarding physical activity, symptomatic dizziness, or other covariates were excluded.

#### Participants’ physical activity and symptomatic dizziness

Each NHANES participant completed a comprehensive set of questionnaires, including the Physical Activity Questionnaire (PAQ) and the Balance Questionnaire (BAQ). The PAQ provides detailed information regarding physical activities, including exercise, sports, and recreational physical hobbies, as well as the intensity of these activities [10]. The PAQ asks about the frequency and intensity of activities over the past 30 days, including whether participants engaged in at least 10 minutes of vigorous activity that resulted in substantial sweating or a significant increase in breathing or heart rate. It also asks whether they participated in at least 10 minutes of moderate activity that caused only slight perspiration or a mild-to-moderate increase in breathing or heart rate. Based on the responses, participants were divided into the following three groups according to levels of physical activity, Group 1 (sedentary: almost no engagement in any form of aerobic or anaerobic exercise in the past 30 days), Group 2 (moderate: at least 10 minutes of moderate-intensity physical activity in the past 30 days, which results in light perspiration or a minor to moderate rise in heart rate and breathing rate), and Group 3 (vigorous: engaging in at least 10 minutes of vigorous-intensity exercise over the last 30 days, which leads to substantial sweating or a pronounced increase in both breathing and heart rates) [11, 12]. This classification provides a nuanced understanding of participants’ physical activity habits and their potential impact on health outcomes. We determined the presence of dizziness, balance difficulties, or falling challenges by analyzing their responses to the BAQ question: “Have you experienced dizziness, balance problems, or difficulty with falling in the past 12 months?” [1].

#### Covariates

This study meticulously assessed a spectrum of potential covariates, including factors such as age, sex, race, education, marital status, poverty income ratio, smoking habits, alcohol consumption, body mass index (BMI), and the presence of chronic conditions such as hypertension, diabetes, coronary heart disease (CHD), stroke, and migraine, as well as levels of hemoglobin, triglycerides, total cholesterol, and C-reactive protein. Ethnicity was categorized as non-Hispanic white, non-Hispanic black, Mexican American, or other. Marital status was defined as married, cohabiting with a partner, or living alone. Education level was stratified into categories of less than 9 years, 9–12 years, or more than 12 years of schooling. Smoking status was classified according to preceding literature definitions: never smokers(smoked less than 100 cigarettes), current smokers, or former smokers(quit smoking after smoking more than 100 cigarettes) [13]. BMI was calculated using a standard method based on individual weight and height. Dietary recall interviews were conducted prior to the Mobile Examination Center interviews. Past medical conditions were evaluated using questionnaires regarding blood pressure, diabetes, and other diseases such as stroke and CHD. This comprehensive approach ensures a nuanced understanding of the interplay between various factors and their potential impact on health outcomes.

#### Statistical analysis

This analysis is a secondary examination of a publicly accessible dataset. Categorical variables are expressed as proportions (%), while continuous variables are described using means (standard deviation, SD) or medians (interquartile range, IQR), depending on the distribution. To compare differences across groups, one-way analysis of variance was employed for normally distributed data, the Kruskal-Wallis test for skewed distributions, and chi-square tests for categorical variables.

Logistic regression models were employed to assess the odds ratios (OR) and 95% confidence intervals (95% CI) for the association between physical activity and symptomatic dizziness. Model I was adjusted for sociodemographic factors such as sex, age, race/ethnicity, education level, marital status, and family income. Model II includes adjustments for comorbid chronic conditions. Model III was fully adjusted for sociodemographic factors, lifestyle behaviors such as smoking and drinking, BMI, and the presence of chronic conditions including hypertension, diabetes, CHD, stroke, and migraine, as well as levels of hemoglobin, triglycerides, total cholesterol, and C-reactive protein. This comprehensive modeling approach ensures a robust assessment of the association while controlling for a wide array of influential factors.

Additionally, the potential variability in the relationship between physical activity and symptomatic dizziness was evaluated, encompassing variables such as sex, age (< 65 years versus ≥ 65 years), BMI (< 25 kg/m^2^ versus ≥ 25 kg/m^2^), hypertension (yes or no), diabetes (yes or no), and migraine (yes or no). Heterogeneity among subgroups was assessed using multivariate logistic regression, and interactions between subgroups and intensity of physical activity were examined using likelihood ratio tests. This refined approach provides a nuanced understanding of how different demographic and health-related factors influence the association between physical activity and the prevalence of symptomatic dizziness, offering insights that can inform targeted interventions and personalized recommendations for individuals across various subgroups.

Because the sample size was based exclusively on the data provided, prior statistical power calculations were not conducted. All analyses were performed using the R statistical software package version 3.3.2 (available from http://www.r-project.org, maintained by The R Foundation, Shanghai, China, as of March 10, 2022) and Free Statistics software version 1.92. Descriptive analysis was performed for the entire participant pool. Significance was ascertained through two-tailed tests, with a p-value threshold of less than 0.05.

## Results

### Study population characteristics

**Fig. 1:**
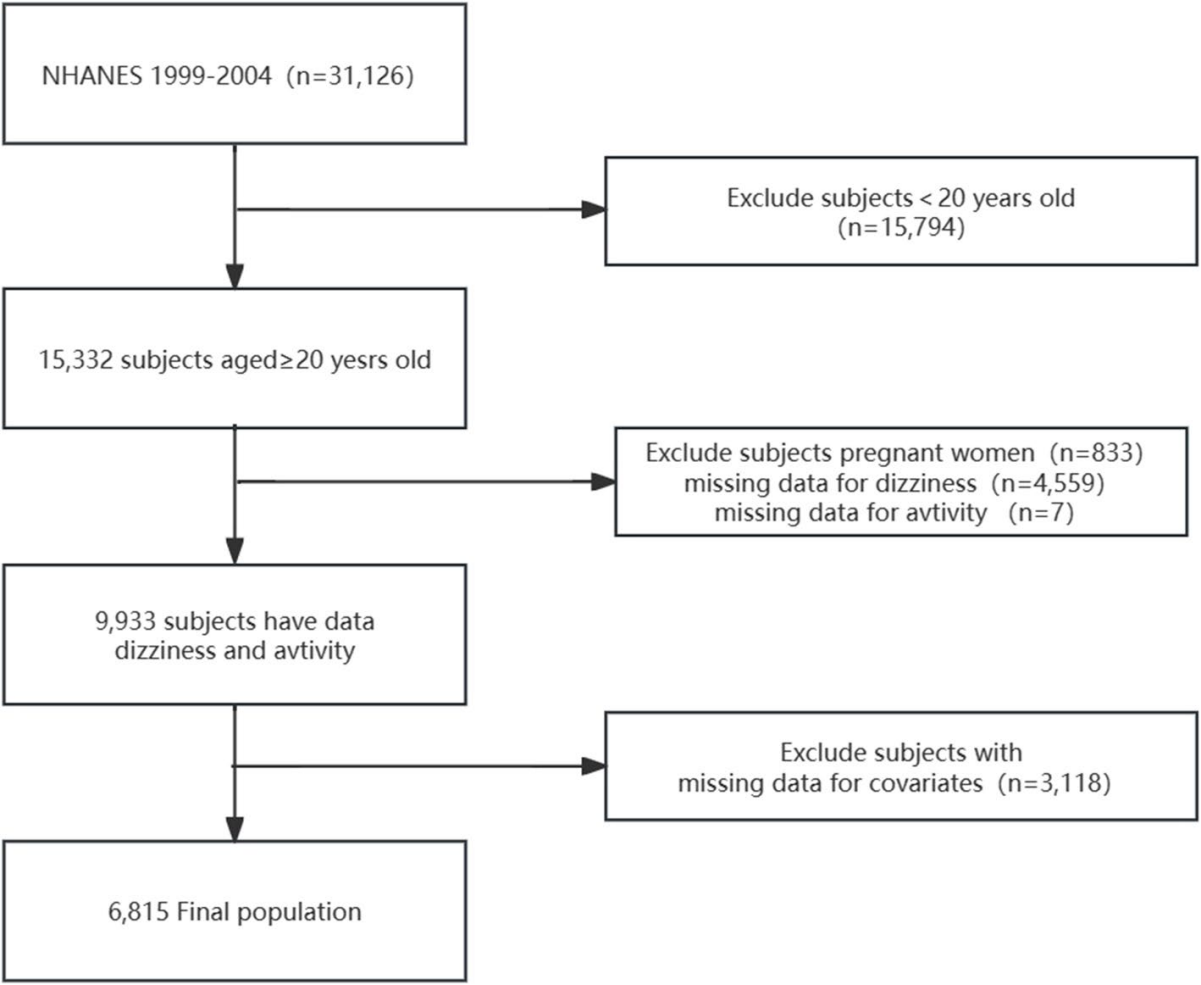
Flowchart of the population included in our final analysis

A total of 31,126 individuals participated in the interviews, 15,794 of whom were under the age of 20. From this pool, we excluded participants who were pregnant (*n*= 833), had incomplete information on dizziness (*n*= 4,559), lacked data on physical activity (*n*= 7), or had missing covariates (*n*= 3,118). As a result, our cross-sectional analysis included 6,815 participants derived from the NHANES data collected between 1999 and 2004. The comprehensive inclusion and exclusion criteria are shown in Fig. 1.

### Baseline characteristics

Table 1 presents the baseline characteristics of all participants according to the presence of symptomatic dizziness. A total of 1,748 people (25.6%) experienced symptomatic dizziness. The mean age of the participants was 60.6 ± 13.3 years, and 3,369 (49.4%) were women. Individuals with symptomatic dizziness tend to be older, female, married or living with a partner, non-Hispanic white, never smokers, alcohol drinkers, have a higher education level, a moderate family income, engage in less physical activity, and have a higher prevalence of hypertension, diabetes, CHD, stroke, and migraine.

**Table 1:** Baseline characteristics of the study population by symptomatic dizziness

Variables	Total (*n* = 6815)	Symptomatic dizziness		*P*-value
		no(*n* = 5067)	yes (*n* = 1748)	
Sex, n (%)				< 0.001
Male	3446 (50.6)	2719 (53.7)	727 (41.6)	
Female	3369 (49.4)	2348 (46.3)	1021 (58.4)	
Age(year),Mean(SD)	60.6 ± 13.3	59.6 ± 13.1	63.6 ± 13.7	< 0.001
Race/ethnicity,n (%)				< 0.001
Non-Hispanic white	3801 (55.8)	2796 (55.2)	1005 (57.5)	
Non-Hispanic black	1192 (17.5)	926 (18.3)	266 (15.2)	
Mexican American	1417 (20.8)	1069 (21.1)	348 (19.9)	
Others	405 (5.9)	276 (5.4)	129 (7.4)	
Education level(year), n (%)				< 0.001
< 9	1228 (18.0)	867 (17.1)	361 (20.7)	
9–12	1052 (15.4)	734 (14.5)	318 (18.2)	
> 12	4535 (66.5)	3466 (68.4)	1069 (61.2)	
Marital status, n (%)				< 0.001
Married or living with a partner	4462 (65.5)	3472 (68.5)	990 (56.6)	
Living alone	2353 (34.5)	1595 (31.5)	758 (43.4)	
Family income,n (%)				< 0.001
Low	1773 (26.0)	1150 (22.7)	623 (35.6)	
Medium	2602 (38.2)	1891 (37.3)	711 (40.7)	
High	2440 (35.8)	2026 (40)	414 (23.7)	
Smoking, n (%)				0.028
Never	3174 (46.6)	2408 (47.5)	766 (43.8)	
Current	1270 (18.6)	927 (18.3)	343 (19.6)	
Former	2371 (34.8)	1732 (34.2)	639 (36.6)	
Alcohol consumption, n (%)	4554 (66.8)	3509 (69.3)	1045 (59.8)	< 0.001
Physical activity,n (%)				< 0.001
Group1	3185 (46.7)	2199 (43.4)	986 (56.4)	
Group2	2114 (31.0)	1622 (32)	492 (28.1)	
Group3	1516 (22.2)	1246 (24.6)	270 (15.4)	
Body mass index(kg/m^2^), Mean(SD)	28.7 ± 6.0	28.7 ± 5.8	28.8 ± 6.5	0.691
Hypertension, n (%)	2494 (36.6)	1637 (32.3)	857 (49)	< 0.001
Diabetes, n (%)	958 (14.1)	600 (11.8)	358 (20.5)	< 0.001
Coronary heart disease, n (%)	468 (6.9)	305 (6)	163 (9.3)	< 0.001
Stroke, n (%)	310 (4.5)	152 (3)	158 (9)	< 0.001
Migraine, n (%)	1176 (17.3)	670 (13.2)	506 (28.9)	< 0.001
Hemoglobin (g/dl), Mean(SD)	14.3 ± 1.5	14.4 ± 1.5	14.1 ± 1.5	< 0.001
Triglyceride (mg/dl), Median (IQR)	123.0 (85.0, 181.0)	121.0 (84.0, 180.0)	127.0 (89.0, 188.0)	< 0.002
Total cholesterol(mg/dl), Mean (SD)	208.8 ± 41.7	209.4 ± 41.8	207.1 ± 41.5	0.045
C-reactive protein(mg/dl), Median (IQR)	0.2 (0.1, 0.6)	0.2 (0.1, 0.5)	0.3 (0.1, 0.7)	< 0.001

*Abbreviations*: *Group1 *sedentary(Almost no engagement in any form of aerobic or anaerobic exercise in the past 30 days), *Group2 *moderate (At least 10 minutes of moderate-intensity physical activity in the past 30 days, which results in light perspiration or a minor to moderate rise in heart rate and breathing rate), *Group3 *vigorous (Engaging in at least 10 minutes of vigorous-intensity exercise over the last 30 days, which leads to substantial sweating or a pronounced increase in both breathing and heart rates)

### Relationship between physical activity and symptomatic dizziness

Univariate analysis showed that sex, age, race, education level, marital status, family income, smoking status, alcohol consumption, physical activity, hypertension, diabetes, CHD, stroke, migraine, hemoglobin level, and C-reactive protein level were associated with symptomatic dizziness (Table 2). After adjusting for potential confounders, a significant inverse association was observed between physical activity and symptomatic dizziness. In Model III, the adjusted odds ratios (OR) for Group 2 (moderate) and Group 3 (vigorous) compared to Group 1 (sedentary) were 0.76 (95% CI 0.66–0.87, *p*< 0.001) and 0.76 (95% CI 0.64–0.90, *p*< 0.001), respectively (Table 3). This indicates that adults with moderate and vigorous physical activity had a 24% lower prevalence of symptomatic dizziness than those in the sedentary group.

**Table 2:** Association of covariates and Symptomatic dizziness risk

Variable	OR(95%CI)	*p*-value
Sex		
Male	1(reference)	
Female	1.63 (1.46 ~ 1.82)	< 0.001
Age(years)	1.02 (1.02 ~ 1.03)	< 0.001
Race/ethnicity		
Non-Hispanic white	1(reference)	
Non-Hispanic black	0.83 (0.68 ~ 0.93)	0.004
Mexican American	0.91 (0.79 ~ 1.04)	0.168
Others	1.30 (1.04 ~ 1.62)	0.02
Education level(year)		
< 9	1(reference)	
9–12	1.04 (0.87 ~ 1.25)	0.665
> 12	0.74 (0.64 ~ 0.85)	< 0.001
Marital status		
Married or living with a partner	1(reference)	
Living alone	1.65 (1.49 ~ 1.86)	< 0.001
Family income		
Low	1(reference)	
Medium	0.69 (0.61 ~ 0.79)	< 0.001
High	0.38 (0.33 ~ 0.44)	< 0.001
Smoking		
Never	1(reference)	
Current	1.16 (1 ~ 1.35)	0.046
Former	1.16 (1.03 ~ 1.31)	0.017
Alcohol consumption		
No	1(reference)	
Yes	0.66(0.59 ~ 0.74)	< 0.001
Physical activity		
Group1	1(reference)	
Group2	0.68 (0.60 ~ 0.77)	< 0.001
Group3	0.48 (0.42 ~ 0.56)	< 0.001
Body mass index(kg/m^2^)	1 (0.99 ~ 1.01)	0.691
Hypertension		
No	1(reference)	
Yes	2.02 (1.80 ~ 2.25)	< 0.001
Diabetes		
No	1(reference)	
Yes	1.92 (1.66 ~ 2.21)	< 0.001
Coronary heart disease		
No	1(reference)	
Yes	1.61 (1.32 ~ 1.96)	< 0.001
Stroke		
No	1(reference)	
Yes	3.21(2.55 ~ 4.04)	< 0.001
Migraine		
No	1(reference)	
Yes	2.67 (2.34 ~ 3.05)	< 0.001
Hemoglobin (g/dl)	0.90(0.86 ~ 0.93)	< 0.001
Triglyceride (mg/dl)	1 (1 ~ 1)	0.95
Total cholesterol(mg/dl)	1 (1 ~ 1)	0.045
C-reactive protein(mg/dl)	1.12(1.06 ~ 1.18)	< 0.001

*Abbreviations*: *Group1 *sedentary(Almost no engagement in any form of aerobic or anaerobic exercise in the past 30 days), *Group2 *moderate (At least 10 minutes of moderate-intensity physical activity in the past 30 days, which results in light perspiration or a minor to moderate rise in heart rate and breathing rate), *Group3 *vigorous (Engaging in at least 10 minutes of vigorous-intensity exercise over the last 30 days, which leads to substantial sweating or a pronounced increase in both breathing and heart rates), *OR *odds ratio, *95% CI *95% confidence interval

**Table 3:** Association between physical activity and symptomatic dizziness

Physical activity	n.total	n.event_%	Crude Model		Model I		Model II		Model III	
			OR (95%CI)	*P*-value	OR (95%CI)	*P*-value	OR (95%CI)	*P*-value	OR (95%CI)	*P*-value
Group1	3185.0	986 (31)	1(Ref)		1(Ref)		1(Ref)		1(Ref)	
Group2	2114.0	492 (23.3)	0.68 (0.60 ~ 0.77)	< 0.001	0.74 (0.65 ~ 0.85)	< 0.001	0.76 (0.66 ~ 0.87)	< 0.001	0.76 (0.66 ~ 0.87)	< 0.001
Group3	1516.0	270 (17.8)	0.48 (0.42 ~ 0.56)	< 0.001	0.69 (0.58 ~ 0.81)	< 0.001	0.75 (0.63 ~ 0.89)	0.001	0.76 (0.64 ~ 0.90)	0.001
P for trend				< 0.001		< 0.001		< 0.001		< 0.001

Group1 as sedentary(Almost no engagement in any form of aerobic or anaerobic exercise in the past 30 days)

Group2 as moderate (At least 10 minutes of moderate-intensity physical activity in the past 30 days, which results in light perspiration or a minor to moderate rise in heart rate and breathing rate)

Group3 as vigorous (Engaging in at least 10 minutes of vigorous-intensity exercise over the last 30 days, which leads to substantial sweating or a pronounced increase in both breathing and heart rates)

Model I: adjusted for sociodemographic variables (sex,age, race/ethnicity, education level,marital status and family income)

Model II:adjusted for model I + hypertension,diabetes,coronary heart disease,stroke,migraine

Model III: adjusted for Model II+hemoglobin,triglycerides,total cholesterol,c-reactive protein,smoking,alcohol consumption,body mass index

*OR* odds ratio, *95% CI* 95% confidence interval

### Stratified analyses based on additional variables

**Fig. 2:**
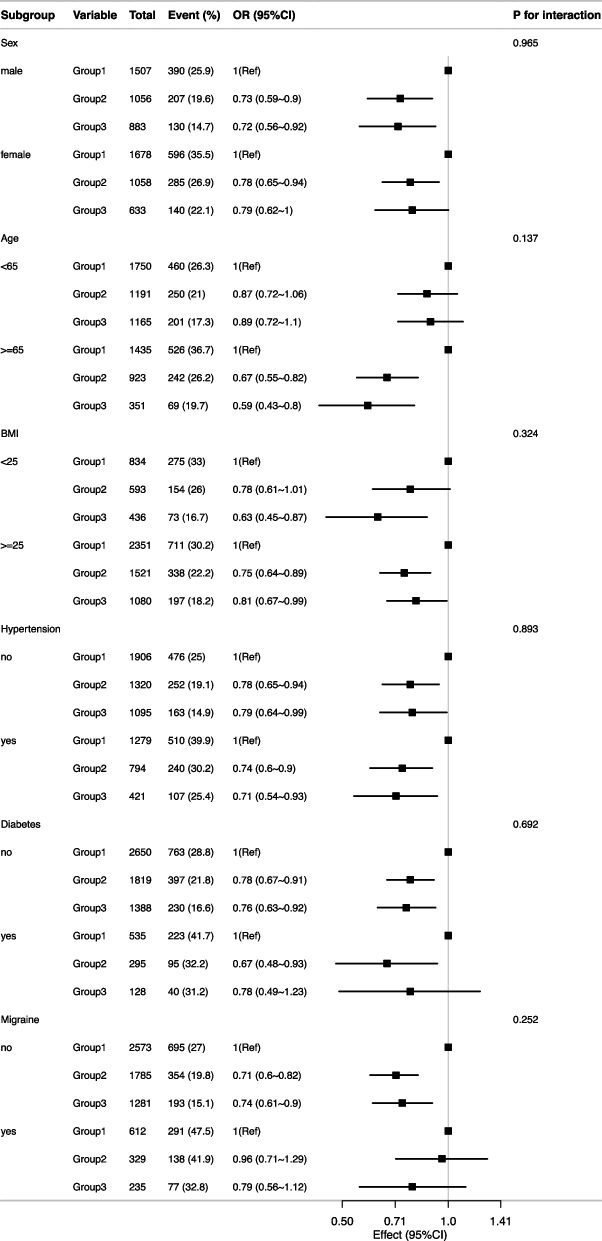
Associations between physical activity and symptomatic dizziness in different subgroups. Except for the stratification component itself, each stratification factor was adjusted for sex, age, race/ethnicity, education level, marital status, family income, hypertension, diabetes, coronary heart disease, stroke, migraine, hemoglobin, triglycerides, total cholesterol, c-reactive protein, smoking, alcohol consumption, body mass index. OR, odds ratio; 95% CI, 95% confidence interval. BMI, body mass index

Stratified analyses were performed on several subgroups to assess the potential impact of physical activity on symptomatic dizziness. No significant interactions were found in any subgroup when stratified by sex, age, BMI, hypertension, diabetes, or migraine (Fig. 2).

## Discussion

In this study, we found an inverse correlation between physical activity and the prevalence of symptomatic dizziness in American adults. Even after stratified analysis, a association between physical activity and a reduced prevalence of symptomatic dizziness persisted. Studies [10, 11, 14, 15] related to this topic report that as physical activity increases, the incidence of diabetes, hypertension, osteoarthritis, and sleep disorders decreases. Other studies have shown a negative correlation between daily physical activity and thyroid hormone levels, inflammation, and immune system markers in both men and women [16]. However, research directly linking physical activity to symptomatic dizziness is scarce.

NHANES offers a unique opportunity to investigate the potential association between physical activity and symptomatic dizziness. This assessment was conducted with comprehensive adjustments for numerous covariates and a range of stratified analyses, thereby providing a thorough and well-controlled examination of the relationship.

The main strength of this study was the use of a considerable sample of national representatives. In the present study, the correlation between physical activity and symptomatic dizziness in adults was estimated using logistic regression. Furthermore, physical activity is painless and helps ameliorate various diseases.

Physical activity can reduce dizziness through several mechanisms. Improved blood circulation: physical activity enhances cardiovascular function and increases the efficiency of blood and oxygen delivery, which can reduce dizziness caused by inadequate blood supply [17, 18]. Enhanced balance ability: engaging in balance and coordination exercises improves body stability, particularly benefiting individuals experiencing dizziness related to vestibular system issues [19, 20]. Facilitation of vestibular adaptation: specific vestibular rehabilitation exercises assist the brain in adapting to and alleviating dizziness resulting from vestibular dysfunction [4, 20, 21]. Reduction of stress and anxiety: physical activity stimulates the release of endorphins, which enhance mood and help mitigate dizziness induced by psychological factors [11, 22]. Promotion of overall health: regular physical activity helps maintain a healthy body weight and metabolic function, indirectly reducing the risk of dizziness associated with various health conditions [15, 16, 23]. Collectively, these mechanisms contribute to a decrease in the frequency and severity of dizziness.

However, this study has some limitations. Firstly, owing to the cross-sectional design of this study, a causal relationship between physical activity and symptomatic dizziness could not be determined. Our study relies on self-reported data from participants in the NHANES database, which may introduce recall bias. NHANES uses questionnaires to collect information on physical activity and symptomatic dizziness. However, such self-reported data lack clinical details in key dimensions such as the frequency, duration, severity, and triggers of symptoms [1, 24]. Consequently, the term “dizziness” may encompass a heterogeneous group of conditions, including vestibular, cardiovascular, and psychogenic causes. This heterogeneity could lead to misclassification of the outcome and may potentially dilute the observed associations. Moreover, self-reports of physical activity and dizziness symptoms may be distorted due to memory bias. For instance, participants’ inaccurate recollection of past activities or symptoms may affect the objectivity of the data. Secondly, the self-reported data on dizziness lack clinical granularity in key aspects, and potential confounding factors such as sedentary behavior have not been adequately assessed. NHANES lacks information on diseases that may cause dizziness, such as inner ear disorders or central nervous system diseases (e.g., Parkinson’s disease). Additionally, it does not include data on dizziness-related medication use, work stress [25], working hours [26], or tendencies towards anxiety or depression [27], all of which are potential confounding factors that may introduce selection bias in dizziness. Recent studies have indicated that sedentary behavior can independently affect health outcomes related to dizziness [28, 29]. Future research with larger sample sizes is needed to further explore the relationship between physical activity of varying intensities and symptomatic dizziness, and to elucidate the underlying mechanisms. Thirdly, when conducting subgroup analyses (e.g., among older individuals, females, or those with chronic diseases), the statistical power is significantly reduced, which may affect the ability to detect interactions. Although our study did not find any interactions, this may be attributable to the limitations of the sample size. We plan to further explore this issue in future research.

## Conclusion

There was a negative association between physical activity and the prevalence of symptomatic dizziness in the adult population in the United States. The results of this study suggest that paying attention to increased physical activity may help reduce symptomatic dizziness.

## Data Availability

No datasets were generated or analysed during the current study.

## Abbreviations

NHANES: National Health and Nutrition Examination Survey
PAQ: Physical Activity Questionnaire
BAQ: Balance Questionnaire
BMI: Body Mass Index
CHD: Coronary Heart Disease
